# Development and Application of a Machine Learning Approach to Assess Short-term Mortality Risk Among Patients With Cancer Starting Chemotherapy

**DOI:** 10.1001/jamanetworkopen.2018.0926

**Published:** 2018-07-27

**Authors:** Aymen A. Elfiky, Maximilian J. Pany, Ravi B. Parikh, Ziad Obermeyer

**Affiliations:** 1 Department of Medical Oncology, Dana-Farber Cancer Institute, Boston, Massachusetts; 2 Brigham and Women’s Hospital, Boston, Massachusetts; 3 Harvard Medical School, Boston, Massachusetts; 4 Division of Hematology and Oncology, Perelman School of Medicine, University of Philadelphia, Philadelphia, Pennsylvania; 5 Leonard Davis Institute of Health Economics, Philadelphia, Pennsylvania

## Abstract

**Importance:**

Patients with cancer who die soon after starting chemotherapy incur costs of treatment without the benefits. Accurately predicting mortality risk before administering chemotherapy is important, but few patient data–driven tools exist.

**Objective:**

To create and validate a machine learning model that predicts mortality in a general oncology cohort starting new chemotherapy, using only data available before the first day of treatment.

**Design, Setting, and Participants:**

This retrospective cohort study of patients at a large academic cancer center from January 1, 2004, through December 31, 2014, determined date of death by linkage to Social Security data. The model was derived using data from 2004 through 2011, and performance was measured on nonoverlapping data from 2012 through 2014. The analysis was conducted from June 1 through August 1, 2017. Participants included 26 946 patients starting 51 774 new chemotherapy regimens.

**Main Outcomes and Measures:**

Thirty-day mortality from the first day of a new chemotherapy regimen. Secondary outcomes included model discrimination by predicted mortality risk decile among patients receiving palliative chemotherapy, and 180-day mortality from the first day of a new chemotherapy regimen.

**Results:**

Among the 26 946 patients included in the analysis, mean age was 58.7 years (95% CI, 58.5-58.9 years); 61.1% were female (95% CI, 60.4%-61.9%); and 86.9% were white (95% CI, 86.4%-87.4%). Thirty-day mortality from chemotherapy start was 2.1% (95% CI, 1.9%-2.4%). Among the 9114 patients in the validation set, the most common primary cancers were breast (21.1%; 95% CI, 20.2%-21.9%), colorectal (19.3%; 95% CI, 18.5%-20.2%), and lung (18.0%; 95% CI, 17.2%-18.8%). Model predictions were accurate for all patients (area under the curve [AUC], 0.940; 95% CI, 0.930-0.951). Predictions for patients starting palliative chemotherapy (46.6% of regimens; 95% CI, 45.8%-47.3%), for whom prognosis is particularly important, remained highly accurate (AUC, 0.924; 95% CI, 0.910-0.939). To illustrate model discrimination, patients were ranked initiating palliative chemotherapy by model-predicted mortality risk, and observed mortality was calculated by risk decile. Thirty-day mortality in the highest-risk decile was 22.6% (95% CI, 19.6%-25.6%); in the lowest-risk decile, no patients died. Predictions remained accurate across all primary cancers, stages, and chemotherapies, even for clinical trial regimens that first appeared in years after the model was trained (AUC, 0.942; 95% CI, 0.882-1.000). The same model also performed well for prediction of 180-day mortality (AUC for all patients, 0.870 [95% CI, 0.862-0.877]; highest- vs lowest-risk decile mortality, 74.8% [95% CI, 72.7%-77.0%] vs 0.2% [95% CI, 0.01%-0.4%]). Predictions were more accurate than estimates from randomized clinical trials of individual chemotherapies or the Surveillance, Epidemiology, and End Results data set.

**Conclusions and Relevance:**

A machine learning algorithm using electronic health record data accurately predicted short-term mortality among patients starting chemotherapy. Further research is necessary to determine the generalizability and feasibility of applying this algorithm in clinical settings.

## Key Points

**Question:**

Can a machine learning algorithm applied to electronic health record data predict patients’ short-term risk of death at the time that they begin chemotherapy?

**Findings:**

In this cohort study of 26 946 patients with cancer starting 51 774 discrete chemotherapy regimens, those at high risk of 30-day mortality were accurately identified across palliative and curative chemotherapy regimens and many types and stages of cancer. The algorithm was more accurate than predictions based on randomized clinical trials or population-based registry data.

**Meaning:**

A machine learning algorithm accurately identified individuals at high risk of short-term mortality and may help to guide patient and physician decisions about chemotherapy initiation and advance care planning.

## Teaser

This cohort study describes and applies a machine learning model to predict short-term mortality in a general oncology cohort of patients starting new chemotherapy, using only data available before the first day of treatment.

## Introduction

Chemotherapy lowers the risk of recurrence in early-stage cancers and can improve survival and symptoms in later-stage disease. Balancing these benefits against chemotherapy’s considerable risks is challenging. Increasing evidence suggests that chemotherapy is too often started too late in the cancer disease trajectory,^1,2,3,4^ and many patients die soon after initiating treatment. These patients experience burdensome symptoms without many of the potential benefits of chemotherapy.^5^ National organizations now track the proportion of patients who die within 2 weeks of receiving chemotherapy as a marker of poor quality of care,^6,7^ and this number has been increasing rapidly.^1,8^

A key factor underlying these trends is the difficulty of accurately identifying the risk of serious adverse events, especially death, before initiating chemotherapy. Adverse effects of chemotherapy are variable, and the influence of comorbidities is complex; thus, the risk calculus of administering chemotherapy is challenging.^9,10,11,12,13^ Cognitive biases also lead to underestimation of the risk of death,^14,15^ particularly in patients with metastatic cancer,^16,17^ who often believe that their disease is curable.^18,19^ Physicians do not accurately estimate prognosis in patients with cancer,^20,21^ and overly optimistic estimates can influence patients’ chemotherapy decisions.^22,23,24,25,26,27^

To estimate mortality before initiation of chemotherapy, physicians may reference randomized clinical trial (RCT) data for individual regimens or population-level data such as the Surveillance, Epidemiology, and End Results (SEER) data set to obtain mortality risk by age, sex, and primary cancer.^14,28^ Although informative, these tools provide mortality estimates for broad populations of patients and often do not accurately estimate a specific individual’s mortality. Individualized decision support tools exist^29^ but require a substantial investment of time and resources; these tools require clinicians to collect and enter data not readily available in existing records, which limits the number of variables that can be used and adds complexity to workflows.

There is considerable enthusiasm for the role of advanced algorithms to improve prediction; just as modern electronic health records (EHRs) pull complex data for clinicians to use in real time, algorithms could pull and process these data in parallel, presenting accurate probability forecasts to clinicians and patients.^30^ However, little evidence suggests that such algorithms can provide meaningful inputs to clinical decision making in cancer or elsewhere.

New chemotherapy is a critical event in the disease trajectory of cancer, and objective predictions of short-term mortality at this time could be useful to physicians and patients in several ways. Accurate forecasts of the risks of mortality and adverse events could inform discussions of risks and benefits of chemotherapy, particularly for patients undergoing palliative chemotherapy, and could help guide important decisions regarding advance care planning and palliative care consultation. In this study, we developed and applied a machine learning algorithm to predict near-term mortality risk in a large cohort of patients with cancer starting new chemotherapy regimens.

## Methods

### Study Population

We obtained EHR data for all patients receiving chemotherapy at the Dana-Farber/Brigham and Women’s Cancer Center (DF/BWCC), Boston, Massachusetts, from January 1, 2004, through December 31, 2014. We determined date of death by linking to the Social Security Administration’s Death Master File. We classified patients by primary cancer and presence of distant-stage disease, determined using registry data (for patients diagnosed at DF/BWCC) and *International Classification of Diseases, Ninth Revision* (*ICD-9*) codes for metastases (for patients not diagnosed at DF/BWCC or who did not have registry data and to identify progression to distant-stage disease in those previously diagnosed at DF/BWCC).^31^ Although diagnosis codes have limitations for determination of cancer stage, they are generally believed to provide reliable identification of the presence or the absence of distant-stage disease.^32^ Our study followed the transparent reporting of a multivariable prediction model for individual prognosis or diagnosis (TRIPOD) checklist for prediction model development and validation (eMethods in the Supplement).The institutional review boards of Dana-Farber Cancer Institute and Partners HealthCare, Boston, approved this study and granted a waiver of informed consent from study participants.

### Statistical Analysis

#### Outcomes

Data were analyzed from June 1 through August 1, 2017. Our primary outcome was death within 30 days of starting new systemic chemotherapy regimens. Secondary outcomes were 30-day mortality in prespecified subgroups of interest (described later) and overall 180-day mortality. We constructed our data set at the patient–chemotherapy regimen level, such that each regimen was a new observation.

#### Model Performance

Machine learning models have the potential to overfit or produce overly optimistic estimates of model performance based on spurious correlations in development data. We thus report results only in an independent validation set, which played no role in model development; as such, overfitting would only lead to poorer model performance in the validation set. Specifically, we used data from 2004 through 2011 for model derivation and data from 2012 through 2014 for model validation. Because our data set was constructed at the patient–chemotherapy regimen level, observations describing different chemotherapy regimens in the same patient are not independent. For patients whose observations appeared before and after January 1, 2012, we randomly assigned all observations from a given patient to the derivation or the validation set, so that no patient appeared in both sets.

#### Statistical Tests

Our primary measure of model performance was the area under the receiver operating characteristic curve (AUC),^33^ which we calculated by comparing the mortality probability estimate from the machine learning model with observed mortality. We calculated 95% CIs of the AUC following the method of DeLong et al.^34^ We report AUC overall and in subgroups of clinical interest, notably age, sex, race/ethnicity, distant-stage disease, individual primary cancers, chemotherapy lines and regimens, and chemotherapy intent (palliative vs curative, identified by the treating physician and recorded as an EHR flag). To benchmark against existing prognostic models, we obtained 1-year mortality estimates from large RCTs of specific chemotherapy regimens and from the SEER program for available subgroups of patients. To give more clinically relevant metrics of predictive accuracy, we also present mortality rates in given deciles of model-predicted risk, typically highest and lowest. When presenting variable summary statistics, we report CIs for means and proportions and the first and third quartiles for medians.

#### Predictors

To transform raw EHR data into variables usable in a prediction model, we first pulled all data from the 1-year period ending the day before chemotherapy initiation (we did not drop patients based on absence of data during this period). Raw data were aggregated into 23 641 potential predictors in the following categories: demographics, prescribed medications, comorbidities and other grouped *ICD-9* diagnoses, procedures,^31^ use of health care resources, vital signs, laboratory results, and terms derived from physician notes using natural language processing.^31^ For each potential predictor, we created the the statistical summary of related EHR entries for 1 month (recent) and for 2 to 12 months (baseline) before chemotherapy initiation. This strategy is outlined in more detail elsewhere.^35^ We also included a variable indexing how many lines of chemotherapy the patient had in total before the current regimen. No data on the current regimen itself (eg, agent, intent) were used in the predictive model. We dropped variables missing in more than 99% of the derivation sample, leaving 5390 predictors in the model.

#### Algorithm

We used gradient-boosted trees, a linear combination of decision trees similar to those used to derive many clinical decision rules to handle large sets of correlated predictors (R package: xgboost).^36,37^ We used 4-fold cross-validation in the development sample to choose model variables (eg, number of trees, variables per tree). The model was configured to produce individual-level probabilities of 30-day mortality. More details are available in eMethods in the Supplement.

#### Missing Values

Each split of each tree in the model (eg, a split on sex) had a default, which is the value (eg, male or female) that occurred more frequently in the training data. Observations with missing values for a given variable were assigned to the default side of the split. This process was effectively a split-specific, probabilistic imputation function that allowed us to avoid excluding observations that were missing data.

#### Model Variance

We decomposed model predictions into the linear contributions of individual variables. We calculated the (linear) sum of squares for individual variables included in the machine learning model and interpreted the residual sum of squares as the contribution of nonlinear terms and interactions used by the model. Because our model used more than 5000 predictors, we chose to report on only a small selection, specifically (1) those that most explained model variance and (2) those identified as predictors of mortality in prior studies.^29,38,39^ Details on our calculation of the model variance explained by individual predictors are in eMethods in the Supplement.

## Results

### Study Population

We identified 26 946 patients who initiated 51 774 discrete chemotherapy regimens from 2004 through 2014; 59.4% had distant-stage disease. Table 1 shows patient characteristics at the time of chemotherapy initiation. Mean patient age was 58.7 years (95% CI, 58.5-58.9 years), 61.1% were female (95% CI, 60.4%-61.9%), and 86.9% were white (95% CI, 86.4%-87.4%). The most common chemotherapy regimens (derivation and validation sets) were carboplatin and paclitaxel (n = 4042), gemcitabine hydrochloride (n = 2185), and albumin-bound paclitaxel (n = 1985); 3.3% of chemotherapy regimens in the validation set (n = 523) were chemotherapy regimens that first appeared in 2012 or later and thus did not appear in the derivation set. Experimental agents not approved by the US Food and Drug Administration constituted 2.2% (n = 343) of all chemotherapy regimens in the validation set.

**Table 1.** Patient Characteristics of Model Derivation and Validation Sets

Variable	Set		
	Derivation (n = 17 832)	Validation (n = 9114)	Difference (n = 8718)
No. of chemotherapy regimens	36 007	15 767	20 240
Age, mean (95% CI), y	58.7 (58.5 to 58.9)	60.7 (60.5 to 61.0)	−2.1 (−2.4 to −1.7)
Female, % (95% CI)	61.1 (60.4 to 61.9)	60.7 (59.7 to 61.7)	0.5 (−0.7 to 1.7)
White, % (95% CI)	86.9 (86.4 to 87.4)	88.3 (87.7 to 89.0)	−1.5 (−2.3 to −0.7)
Inpatient visit, % (95% CI)	43.9 (43.2 to 44.6)	38.0 (37.0 to 38.9)	6.0 (4.7 to 7.2)
Gagne score, median (IQR)	2 (1 to 6)	1 (1 to 6)	−1 (0 to 0)^a^
Cancer type, % (95% CI)			
Breast	23.6 (23.0 to 24.3)	21.1 (20.2 to 21.9)	2.5 (1.5 to 3.6)
Colon and rectum	17.6 (17.1 to 18.2)	19.3 (18.5 to 20.2)	−1.7 (−2.7 to −0.7)
Lung and bronchus	17.7 (17.2 to 18.3)	18.0 (17.2 to 18.8)	−0.3 (−1.3 to 0.7)
Hematologic	7.1 (6.7 to 7.5)	8.4 (7.8 to 8.9)	−1.3 (−2.0 to −0.6)
Ovary	6.7 (6.3 to 7.0)	7.7 (7.1 to 8.2)	−1.0 (−1.7 to −0.3)
Other	27.8 (27.1 to 28.4)	25.8 (24.9 to 26.7)	2.0 (0.9 to 3.1)
Chemotherapy beyond first line, % (95% CI)	49.0 (48.3 to 49.7)	42.4 (41.4 to 43.4)	6.6 (5.3 to 7.8)
Intent of chemotherapy, % (95% CI)^b^			
Curative	15.7 (15.3 to 16.1)	26.8 (26.1 to 27.5)	−11.1 (−11.9 to −10.3)
Palliative	33.8 (33.3 to 34.3)	46.6 (45.8 to 47.3)	−12.7 (−13.7 to −11.8)

Abbreviation: IQR, interquartile range.

^a^ Calculated by quantile regression.

^b^ Intent of chemotherapy does not add up to 100% owing to missing values, resulting in unknown intent.

### Model Performance

Among the 9114 patients in the validation set, overall 30-day mortality was 2.1% (95% CI, 1.9%-2.4%). The most common primary cancers were breast (21.1%; 95% CI, 20.2%-21.9%), colorectal (19.3%; 95% CI, 18.5%-20.2%), and lung (18.0%; 95% CI, 17.2%-18.8%). The model accurately predicted 30-day mortality for all patients, irrespective of chemotherapy intent (AUC, 0.940; 95% CI, 0.930-0.951). In the subset of patients receiving palliative chemotherapy (46.6% of regimens; 95% CI, 45.8%-47.3%), 30-day mortality was 3.1% (95% CI, 2.7%-3.5%). Prognostic estimates are likely to be particularly important for these patients, and the model also performed well for this situation, with an AUC of 0.924 (95% CI, 0.910-0.939). To illustrate the clinical implications of this accuracy, we used model predictions to individually rank patients by 30-day mortality risk, a commonly used way of stratifying risk groups.^33^ Thirty-day mortality in the highest decile of predicted risk for palliative-intent chemotherapy was 22.6% (95% CI, 19.6%-25.6%), whereas in the lowest-risk decile, no patients died.

**Figure 1.**
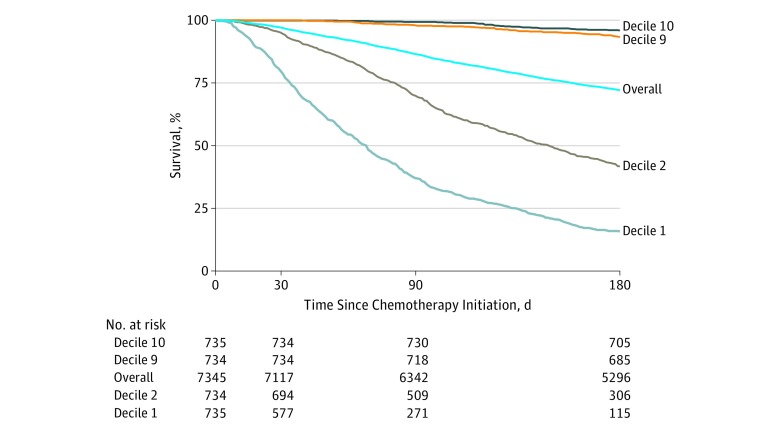
Observed 180-Day Survival From the Initiation of Palliative Chemotherapy Data are stratified by decile of model-predicted mortality risk. Decile 1 denotes the highest predicted risk decile; decile 10, the lowest. Overall denotes overall mean survival among all patients, irrespective of model-predicted risk.

Figure 1 shows observed survival during the 180 days after palliative chemotherapy initiation by decile of model predictions (patients were followed up to 180 days). Overall 180-day mortality among all patients was 18.4% (95% CI, 17.8%-19.0%); for those initiating palliative chemotherapy, 180-day mortality was 27.9% (95% CI, 26.9%-28.9%). Model predictions on 30-day mortality were also accurate predictors of 180-day mortality (AUC, 0.827; 95% CI, 0.817-0.838); in the highest-risk decile, 180-day mortality was 74.8% (95% CI, 72.7%-77.0%) vs 0.2% (95% CI, 0.01%-0.4%) in the lowest-risk decile. Predictions were even more accurate for all patients, irrespective of chemotherapy intent (AUC, 0.870; 95% CI, 0.862-0.877); 180-day survival among these patients is shown in the eFigure in the Supplement.

Table 2 shows model performance for predicting 30-day mortality in additional patient subgroups of interest. The model performed equally well across many kinds of primary cancers, demographic groups, and chemotherapy regimens. In distant-stage disease (mean 30-day mortality, 2.9%; 95% CI, 2.5%-3.2%), 30-day mortality in the highest-risk decile was 22.7% (95% CI, 19.9%-25.6%) vs 0 in the lowest decile (AUC, 0.924; 95% CI, 0.910-0.939). Predictions were accurate even for experimental clinical trial regimens first used from 2012 to 2014 (AUC, 0.942; 95% CI, 0.882-1.000); the derivation model was not exposed to these novel regimens in the training process.

**Table 2.** Model Performance in Selected Subgroups

Subgroup	AUC (95% CI)		No. in Validation Sample
	30-d Mortality	180-d Mortality	
Intent of chemotherapy			
Curative	0.981 (0.970-0.992)	0.892 (0.872-0.912)	4220
Palliative	0.924 (0.910-0.939)	0.827 (0.817-0.838)	7345
Stage			
Distant	0.938 (0.926-0.951)	0.852 (0.842-0.861)	8531
Nondistant	0.936 (0.916-0.955)	0.874 (0.862-0.887)	7236
Cancer			
Breast	0.970 (0.956-0.983)	0.939 (0.928-0.951)	3408
Colon and rectum	0.924 (0.898-0.949)	0.843 (0.827-0.860)	2868
Lung and bronchus	0.916 (0.884-0.949)	0.820 (0.803-0.838)	2822
Ovary	0.956 (0.922-0.989)	0.895 (0.871-0.918)	1632
Hematologic	0.943 (0.902-0.983)	0.819 (0.782-0.856)	1435
Head and neck	0.970 (0.933-1.000)	0.839 (0.798-0.879)	739
Cervix uteri	0.955 (0.906-1.000)	0.888 (0.847-0.929)	599
Prostate	0.936 (0.849-1.000)	0.805 (0.751-0.858)	429
Chemotherapy regimen			
Carboplatin plus paclitaxel	0.940 (0.859-1.000)	0.846 (0.813-0.880)	1297
FOLFOX	0.952 (0.923-0.982)	0.863 (0.833-0.893)	719
Gemcitabine hydrochloride	0.865 (0.789-0.942)	0.992 (0.982-1.000)	547
Pemetrexed plus carboplatin	0.960 (0.939-0.981)	0.807 (0.769-0.845)	518
Paclitaxel	0.989 (0.972-1.000)	0.738 (0.686-0.791)	505
Novel clinical trial agents^a^	0.942 (0.882-1.000)	0.870 (0.817-0.923)	343
Line of chemotherapy			
First	0.941 (0.925-0.956)	0.865 (0.854-0.875)	9114
Subsequent	0.938 (0.924-0.952)	0.864 (0.854-0.874)	6653
Year^b^			
2012	0.951 (0.935-0.966)	0.887 (0.875-0.899)	5169
2013	0.935 (0.914-0.955)	0.869 (0.857-0.882)	4863
2014	0.928 (0.908-0.947)	0.837 (0.824-0.851)	4915
Age group, y			
21-40	0.963 (0.939-0.988)	0.901 (0.874-0.928)	1057
41-60	0.950 (0.935-0.966)	0.893 (0.883-0.903)	6263
61-80	0.936 (0.920-0.951)	0.850 (0.839-0.861)	7626
81-100	0.876 (0.806-0.947)	0.803 (0.768-0.838)	806
Sex			
Male	0.927 (0.910-0.944)	0.840 (0.828-0.852)	5799
Female	0.948 (0.935-0.962)	0.886 (0.877-0.894)	9968
Race/ethnicity			
White	0.940 (0.929-0.951)	0.870 (0.862-0.877)	13 960
Nonwhite	0.941 (0.909-0.972)	0.870 (0.850-0.891)	1807
Insurance			
Private	0.949 (0.937-0.962)	0.886 (0.878-0.895)	10 415
Medicare	0.938 (0.911-0.965)	0.854 (0.837-0.872)	2699
Medicaid	0.945 (0.914-0.976)	0.824 (0.784-0.865)	611
Self-pay	0.932 (0.851-1.000)	0.821 (0.753-0.888)	210

Abbreviations: AUC, area under the receiver operating characteristic curve; FOLFOX, leucovorin calcium (folinic acid), fluorouracil, and oxaliplatin.

^a^ Indicates subgroup of patients receiving new clinical trial regimens first observed during 2012 through 2014 (ie, years of data to which the model was not exposed in the training process).

^b^ Observations in the validation set occurring in 2011 (n = 717) were reassigned to 2012 for the purpose of this Table.

A key question is whether model predictions are accurate enough to be useful across a range of primary cancers, stages of disease, or lines of chemotherapy, which constitute scenarios for which prognoses vary widely. Table 2 thus also presents measures of overall predictive accuracy for first-line chemotherapy (AUC for 30-day mortality, 0.941 [95% CI, 0.925-0.956]; AUC for 180-day mortality, 0.865 [95% CI, 0.854-0.875]) compared with later lines of chemotherapy (AUC for 30-day mortality, 0.938 [95% CI, 0.924-0.952]; AUC for 180-day mortality, 0.864 [95% CI, 0.854-9.874]). eTable 1 in the Supplement presents extended results on model performance for 30- and 180-day mortality across lung, colorectal, breast, and prostate cancers by stage and line of chemotherapy.

### Comparisons With Other Prognostic Estimates

**Figure 2.**
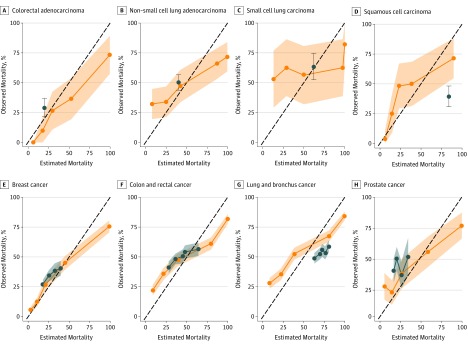
One-Year Mortality After Chemotherapy Initiation A-D, Mortality data from 4 randomized clinical trials^40,41,42,43^ compared with the machine learning model predictions for patients in the validity sample. The randomized clinical trials compared bevacizumab and oxaliplatin,^40^ pemetrexed and carboplatin,^41^ etoposide and carboplatin,^42^ and carboplatin and paclitaxel.^43^ E-H, Mortality estimates from the National Cancer Institute’s Surveillance, Epidemiology, and End Results (SEER) program compared with the machine learning model predictions. The 45° dotted lines denote equivalence of observed and estimated mortality. Orange lines and shaded 95% CIs show observed 1-year mortality against quintiles of model-predicted 30-day mortality risk. Blue lines and shaded 95% CIs show observed 1-year mortality against predictions from 1-year mortality and 95% CI for trial patients taking a given regimen and 1-year mortality estimates from the SEER program, by type, age, sex, and race/ethnicity.

We compared model performance with 2 external sources of mortality estimates, focusing on patients with distant-stage disease. First, we obtained mortality data from 4 RCTs of treatments for colorectal adenocarcinoma, non–small cell lung adenocarcinoma, small cell lung carcinoma, and squamous cell carcinoma of the head and neck.^40,41,42,43^ Figure 2A-D shows observed mortality for patients in our validation sample who started specific chemotherapy regimens for which trial data are available. (We chose to show 1-year mortality because this is the only time window reported consistently in RCTs.) We compared observed mortality with 2 sources of predictions: (1) RCT data (ie, mean 1-year mortality for patients receiving the relevant chemotherapy regimen) and (2) 1-year mortality risk estimates from our model; to generate these, we calculated 1-year mortality in the derivation set for patients in each quintile of model-predicted risk (we could not use raw model predictions because these were designed to predict 30-day mortality). The overall AUC for RCT estimates was 0.555 (95% CI, 0.513-0.598) compared with 0.771 (95% CI, 0.735-0.808) for model-based estimates for these same patients.

We also compared our model predictions of mortality with age-, sex-, race-, and cancer-specific mortality estimates from SEER, restricted to patients with advanced-stage cancers of the lung and bronchus, colon and rectum, breast, and prostate to maximize comparability in populations. Figure 2E-H shows that our model predictions (AUC, 0.810; 95% CI, 0.799-0.822) outperformed SEER estimates (AUC, 0.600; 95% CI, 0.585-0.615) for 1-year mortality. Further details on construction of RCT and SEER estimates are available in the eMethods and eTable 2 in the Supplement, and more detailed comparisons for subgroups are available in eTable 3 in the Supplement.

### Key Predictors

Table 3 shows the distribution of key predictor variables used in the prediction model across risk deciles, as well as the proportion of model variance explained linearly by each variable. In general, key predictors of mortality identified in the literature were markedly different in the highest vs lowest model-predicted risk deciles; these predictors included summed comorbidity score,^39^ age,^38^ failure to thrive, heart rate, and certain laboratory data (eg, C-reactive protein level, white blood cell count, and alkaline phosphatase level).^29^ Of importance, no single variable explained more than 2% of model predictions in linear fashion. Most of the variation in the predictions (86.4%) was not a linear function of any single predictor, indicating that the tree-based model relied heavily on complex nonlinear functional forms and interactions among variables.

**Table 3.** Selected Predictors by Risk Decile and Model Variance Explained

Predictor	Risk Decile			Model Variance Explained, %
	Top	Median	Bottom	
Cancer of the brain and with other nervous system areas, %	6.7	1.6	0.0	1.94
Demographics				
Mean age, y	62.3	62.1	51.9	1.30
Female, %	56.4	60.9	86.9	1.17
Black, %	3.8	3.6	3.4	0.07
Mean comorbidity score^a^	5.14	3.44	2.01	0.03
Prior diagnoses, %				
Ascites	0.31	0.07	0.01	0.39
Mouth disorder	0.02	0.01	0.01	0.25
Nausea and vomiting	0.23	0.09	0.01	0.18
Lower respiratory tract disorders	2.10	1.36	0.16	0.03
Secondary malignant neoplasm	5.57	1.69	0.36	0.01
Failure to thrive	0.05	0.01	0.00	0.01
Nutritional disorders	2.53	1.63	0.54	0.00
Malaise and fatigue	0.22	0.08	0.02	0.00
Medications, %				
Corticosteroids	0.53	0.00	0.00	0.15
Opioids	0.29	0.00	0.00	0.05
Anxiolytics	0.60	0.24	0.18	0.02
Cathartics	0.54	0.00	0.00	0.00
Vital signs^b^				
Maximum pulse, bpm (baseline)	106.1	95.7	87.1	0.37
Maximum weight, kg (baseline)	79.5	80.2	76.8	0.11
Weight, SD, kg (baseline)	3.1	2.1	1.3	0.06
Minimum pulse, bpm (recent)	83.4	72.8	63.0	0.01
Weight change, kg^c^	−3.1	−1.0	0.1	0.00
Laboratory findings^d^				
Maximum C-reactive protein level, mg/L	93.9	65.6	2.2	0.19
Maximum ALT level, U/L	75.9	57.3	24.3	0.07
Maximum AST level, U/L	73.7	54.2	23.8	0.09
Maximum white blood cell count, ×10^3^/μL	13.9	12.4	9.8	0.03
Maximum alkaline phosphatase, IU/L	199.5	128.7	76.5	0.02
Mean lymphocyte count, ×10^3^/μL	1.0	1.3	1.8	0.00
Mean platelet count, ×10^3^/μL	251.8	241.4	265.1	0.00
Ejection fraction, %	54.4	48.0	51.9	0.01
Total linear terms	NA	NA	NA	13.6
Total nonlinear terms and interactions	NA	NA	NA	86.4

Abbreviations: ALT, alanine aminotransferase; AST, aspartate aminotransferase; NA, not applicable.

SI conversion factors: To convert ALT, AST, and alkaline phosphatase to microkatals per liter, multiply by 0.0167; C-reactive protein to nanomoles per liter, multiply by 9.524; and white blood cell count, lymphocyte count, and platelet count to ×10^9^ per liter, multiply by 1.

^a^ Scores range from 0 to 26, with higher scores indicating more comorbidites.

^b^ Baseline and recent denote the value of the predictor (minimum, maximum, or SD, as noted) during the 2 to 12 months and the 1 month before chemotherapy initiation, respectively.

^c^ Indicates the change from the mean value during the 2 to 12 months before to the mean value during the 1 month before chemotherapy initiation.

^d^ Indicates the value of the predictor (minimum or maximum as noted) during the 12 months before chemotherapy initiation.

## Discussion

A machine learning model based on single-center EHR data accurately estimated individual mortality risk in a cohort of patients with cancer at the time of chemotherapy initiation. The model performed well across a range of cancer types, race, sex, and other demographic variables. Mortality estimates were accurate for chemotherapy regimens with palliative and curative intent, for patients with early- and distant-stage cancer, and for patients treated with clinical trial regimens introduced in years after the model was trained. Our model outperformed estimates from RCTs and SEER data, both of which are routinely used by clinicians for quantitative risk predictions.

This model was able to predict mortality with considerable accuracy despite lacking genetic sequencing data, cancer-specific biomarkers, or any detailed information about cancers beyond EHR data. This accuracy underscores the fact that common clinical data elements contained within an EHR (eg, symptoms, comorbidities, prescribed medications, and diagnostic tests) contain surprising amounts of signal for predicting key outcomes in patients with cancer.

One clinically useful advantage of our algorithm is that it would not require manual input from clinicians. Current validated prognostic algorithms require considerable, often difficult input on the part of clinicians. For example, the palliative prognostic score relies on 6 weighted variables; some of these data elements, such as Karnofsky performance status, are not routinely available in the EHR and thus require manual input and calculation.^21^

In contrast, our prognostic algorithm could pull directly from the EHR without manual input. Most inputs to our model are standard data elements in structured format in EHRs, including *ICD-9* and procedure codes and medications. Although our algorithm was developed using a single institution’s data, its inputs are available nearly everywhere with an EHR. In addition, no special infrastructure is required to pull these data from an institution’s data warehouse; in the same way that today’s EHR systems pull a rich set of data from a database to present it to clinicians, an algorithm could pull and process the same data in real time using the processing power on a desktop computer. Although machine learning algorithms require significant computing infrastructure to construct, once derived, they can be applied using minimal computing power already available in any hospital computers running an EHR or even on a smartphone. This application facilitates potential integration into existing clinical systems. Thus, we would not anticipate major technical barriers to implementing this or similar algorithms in any organization’s clinical data to independently validate predictive power from a sample. To this end, code for our algorithm is publicly available (eResults in the Supplement and http://labsysmed.org/wp-content/uploads/2017/02/ChemoMortalityAnalysis.rtf).

Algorithmic predictions such as ours could be useful at several points along the care continuum. They could provide accurate predictions of mortality risk to a clinician or foster shared decision making between the patient and clinician. Short-term estimates of mortality could help clinicians identify patients unlikely to benefit from chemotherapy beyond 30 days and those who may benefit from early palliative care referral, advance care planning, and prompting to get financial and family affairs in order. For patients receiving systemic chemotherapy, an estimate of 30-day mortality risk may be a useful quality indicator of avoidable treatment-associated harm.^44^

### Limitations

This study has several limitations. Our model was built on data from patients treated with chemotherapy and is thus unlikely to be accurate for untreated patients. Second, our treated sample reflects the particular decisions around chemotherapy made by physicians and patients in our training data set. Patients who were eligible for chemotherapy but for some reason did not start it were not included, which could have biased the sample. However, it is likely that the direction of this bias is that prevailing treatment decisions are generally aggressive. In our sample, 62.4% of patients with distant-stage disease received chemotherapy, suggesting that physician recommendations and patient acceptance of those decisions generally lead to initiation of treatment. This finding fits with a large body of evidence suggesting that physicians in a wide range of settings overestimate survival and overuse chemotherapy. Thus, to the extent that our data set has bias, it leads to the inclusion—not exclusion—of patients who otherwise might not have received chemotherapy. As a result, we believe that this bias did not substantially distort validity. If such an algorithm were deployed in a real-world setting, periodic retraining of the model (eg, each year or quarter) would ensure that model predictions reflected contemporaneous chemotherapy decision making. This process would address changing selection into treatment over time and update the model to reflect broader changes in patient populations and chemotherapy technology.

Several significant differences between the 2004-2011 derivation set and the 2012-2014 validation set include age at initiation, race, primary cancer, and prior chemotherapy beyond the first-line treatment. Such differences between derivation and validation sets are expected and intentional: a validation set drawn from later years of data was chosen to reflect the constant evolution of cancer epidemiology and treatment. This process made the prediction task more difficult because algorithms trained on past data cannot always perform well in the future.^45^ However, changes in referral patterns, chemotherapy, and diagnosis patterns are just some of the difficulties associated with algorithms in evolving real-world settings. We are reassured that performance was good despite these and other secular trends.

Although we quantified predictive accuracy in an independent, recent validation set, the only way to truly validate such a model is prospectively. A model trained on pre-2012 data may lose accuracy as novel tumor diagnostics and therapies arise, although the accuracy of predictions for patients starting novel chemotherapies was encouraging in this regard. In addition, this study included data from a single institution. Further validation is required using cohorts from different institutions. Electronic health record data contain a multitude of biases introduced by physician behavior, institutional idiosyncrasies, and software platforms, among other limitations. These limitations can significantly affect the adaptability and relevance of our prediction model to different care settings.

## Conclusions

Our machine learning model accurately predicted mortality risk in patients at the time of chemotherapy initiation. Although we are optimistic that accurate prognostic tools such as this could help to promote value-driven oncology care, the ideal next step would be an RCT of algorithmic estimates at the point of care. To be useful, predictive models must improve decision making in the real world. Thus, rigorous evaluation of predictions’ influence on outcomes is the criterion standard test but one that is often neglected in the literature, which focuses primarily on measuring predictive accuracy rather than real outcomes.

## Notes

Supplement. **eMethods.** Variable Construction, Missing Values, Gradient Boosting, Benchmarking Against SEER Data, and Model Variance **eFigure.** Observed 180-Day Survival From the Initiation of Chemotherapy, Irrespective of Intent **eTable 1.** Top and Bottom Decile Mortality and Model Performance in Selected Subgroup **eTable 2.** Mean Mortality by Primary Cancer, Race, Sex, and Age Group for Distant Stage Disease From 18 SEER Registries **eTable 3.** Model Performance by Category for Machine Learning vs RCT and SEER **eResults.** Code **eReferences**
