# Supplementary material for: Development and Application of a Machine Learning Approach to Assess Short-term Mortality Risk Among Patients With Cancer Starting Chemotherapy
Source: JAMA Netw Open. 2018 Jul 27;1(3):e180926. doi: 10.1001/jamanetworkopen.2018.0926 (PMC6324307; doi:10.1001/jamanetworkopen.2018.0926)
Supplement: Supplement. — eMethods. Variable Construction, Missing Values, Gradient Boosting, Benchmarking Against SEER Data, and Model Variance eFigure. Observed 180-Day Survival From the Initiation of Chemotherapy, Irrespective of Intent eTable 1. Top and Bottom Decile Mortality and Model Performance in Selected Subgroup eTable 2. Mean Mortality by Primary Cancer, Race, Sex, and Age Group for Distant Stage Disease From 18 SEER Registries eTable 3. Model Performance by Category for Machine Learning vs RCT and SEER eResults. Code eReferences [file jamanetwopen-1-e180926-s001.pdf]

Supplementary Online Content

Elfiky AA, Pany MJ, Parikh RB, Obermeyer Z. Development and Application of a Machine Learning Approach to Assess Short-term Mortality Risk Among Patients With Cancer Starting Chemotherapy. *JAMA Netw Open.* 2018;1(3):e180926. doi:10.1001/jamanetworkopen.2018.0926

Supplement. eMethods. Variable Construction, Missing Values, Gradient Boosting, Benchmarking Against SEER Data, and Model Variance

eFigure. Observed 180-Day Survival From the Initiation of Chemotherapy, Irrespective of Intent

eTable 1. Top and Bottom Decile Mortality and Model Performance in Selected Subgroup

eTable 2. Mean Mortality by Primary Cancer, Race, Sex, and Age Group for Distant Stage Disease From 18 SEER Registries

eTable 3. Model Performance by Category for Machine Learning vs RCT and SEER

eResults. Code

eReferences

This supplementary material has been provided by the authors to give readers additional information about their work.

© 2018 Elfiky AA et al. *JAMA Network Open.*

Supplementary Online Content

Elfiky AA, Pany MJ, Parikh RB, Obermeyer Z. Development and Validation of a Machine Learning Approach to Assess Short-term Mortality Risk in Patients With Cancer Starting Chemotherapy. *JAMA Network Open.* Published online July 27, 2018. doi:10.1001/jamanetworkopen.2018.0926

eMethods. Variable Construction, Missing Values, Gradient Boosting, Benchmarking Against SEER Data, and Model Variance
eFigure. Observed 180-Day Survival From the Initiation of Chemotherapy, Irrespective of Intent
eTable 1. Top and Bottom Decile Mortality and Model Performance in Selected Subgroup
eTable 2. Mean Mortality by Primary Cancer, Race, Sex, and Age Group for Distant Stage Disease From 18 SEER Registries
eTable 3. Model Performance by Category for Machine Learning vs RCT and SEER
eResults. Code
References

This supplementary material has been provided by the authors to give readers additional information about their work.

© 2018 Elfiky AA et al. *JAMA Network Open.*

eMethods. Variable Construction, Missing Values, Gradient Boosting, Benchmarking Against SEER Data, and Model Variance

Variable construction: We used electronic health record (EHR) data from all patients receiving chemotherapy at the Dana Farber/Brigham and Women's Cancer Center (DF/BWCC) from 2004–2014 to construct variables for possible inclusion in our prediction model. For patients in this cohort, we obtained all available data on patient demographics, prescribed medications, diagnoses (International Classification of Diseases, Ninth Revision, Clinical Modification; ICD-9-CM codes), procedures (ICD-9-CM and Healthcare Common Procedure Coding System codes), care utilization (*e.g.,* inpatient, outpatient, and emergency encounters), vital signs, and laboratory results. We extracted terms (categorical variables) and values (continuous variables) from physician notes in categories such as ECG reports (*i.e.*, terms such as "atrial fibrillation" or "ST depression" from cardiologist interpretations rather than actual waveforms), functional status, and goals of care. Across EHR categories, we created 23,641 potential predictors over 1) the first month (recent) and 2) months 2 to 12 (baseline) prior to chemotherapy initiation. We empirically evaluated potential predictors for predictive power, and thus inclusion in our mortality algorithm (see section "Gradient boosting" below for more details).

Gradient boosting: We used 4-fold cross-validation in the development sample to empirically choose the model parameters (number of trees, tree depth, and learning rate) that maximized discrimination (area under the receiver-operating characteristic curve, AUC) of a gradient boosted tree algorithm (xgboost[1]) implemented in the R statistical language (version 3.2.3). We chose gradient boosted trees with a logistic objective function to predict mortality risk since this non-parametric algorithm has considerable flexibility in modeling the nonlinearities and high-dimensional interactions inherent in EHR data. Briefly, gradient boosted trees is a machine learning ensemble method that sequentially fits decision trees to the residual of the previous tree in the sequence, resulting in a linear combination of all fitted trees. A comprehensive description of gradient boosted trees can be found elsewhere,[2] and the code we used to implement our model is attached below.[3] After cross-validating and fitting of our model in the derivation set, the most predictive 5,390 variables were retained.

Missing values: For each split of each level of each decision tree (*e.g.*, sex = male or female), the gradient boosted trees model learns a 'default' direction in the derivation set, namely the split to which more observations belong. For instance, if there are more females than males at a particular split on the sex variable in the derivation set, the default direction for that split is female. In the validation set (or any other new set of data), the trained model assigns observations with missing values to the default for the given split. Thus gradient boosted trees impute missing values in a tree split-specific, probabilistic fashion and observations with missing data are retained.

Benchmarking against SEER data: To benchmark against existing prognostic models, we obtained one-year mortality estimates from the National Cancer Institute's Surveillance, Epidemiology, and End Results (SEER) program. For distant-stage disease of the three most common cancers in the validation cohort (lung and bronchus, breast, colon and rectum) as well as prostate cancer, we assigned each patient-visit a one-year mortality estimate from the SEER 18 registries data matched on primary cancer, race, sex, and age group (**eTable 2)**.[4] We then calculated 1) quantiles (quartiles or quintiles, as the spread in SEER mortality estimates allowed) of one-year SEER-estimated mortality as well as 2) quintiles of observed one-year mortality based on our model-predicted 30-day mortality risk by comparing estimates from these two methods to observed mortality status for the one-year period following chemotherapy initiation. We compared both to mean observed one-year mortality. Cancer-specific AUCs are reported in **eTable 3**.

Model variance: In order to make the decomposition of model predictions into linear contributions of individual variables computationally feasible, we first used a least absolute shrinkage and selection operator (lasso)[5] model with mean imputation of missing variables and 5-fold cross-validation to empirically select the most important from the 5,490 model predictors. We then used analysis of variance on coefficient estimates from ordinary least squares regression to calculate the (linear) sum of squares for individual variables. We calculated 'model variance explained' as the proportion of individual variable model sum of squares to overall model sum of squares. We interpreted the residual sum of squares as the contribution of non-linear terms and interactions used by the model. As mentioned in the manuscript,

© 2018 Elfiky AA et al. *JAMA Network Open.*

we can report on only a small selection of our model's more than 5000 predictors, and specifically chose 1) those that most explained model variance, and 2) those identified as predictors of mortality in prior studies.

eFigure. Observed 180-Day Survival From the Initiation of Chemotherapy, Irrespective of Intent

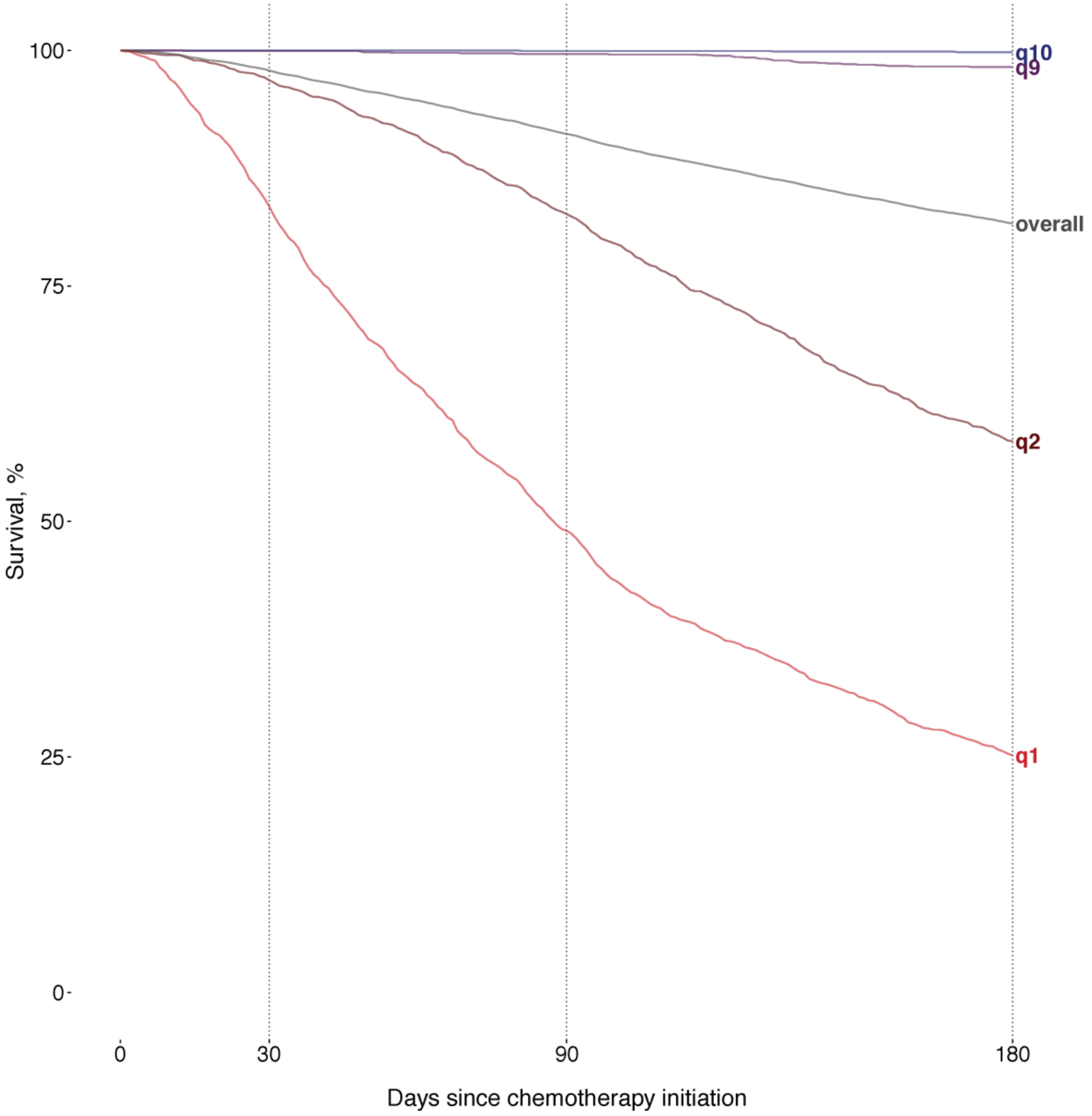

No. at risk				
q10	***1,577***	***1,577***	***1,576***	***1,574***
q9	***1,577***	***1,576***	***1,571***	***1,549***
overall	***15,767***	***15,430***	***14,370***	***12,869***
q2	***1,577***	***1,527***	***1,303***	***922***
q1	***1,577***	***1,316***	***773***	***397***

© 2018 Elfiky AA et al. *JAMA Network Open.*

Note: *q1* denotes the highest predicted risk decile, *q2* the second highest, *q9* the second lowest, and *q10* the lowest. *overall* is baseline survival without risk stratification.

© 2018 Elfiky AA et al. *JAMA Network Open.*

eTable 1. Top and Bottom Decile Mortality and Model Performance in Selected Subgroup

	30-day mortality, % (95% CI)			180-day mortality, % (95% CI)			
**variable**	**top decile**	**bottom decile**	**AUC (95% CI)**	**top decile**	**bottom decile**	**AUC (95% CI)**	**validation sample size**
Intent of chemotherapy							
Curative	5·4 (3·3 to 7·6)	0.0 (0.0 to 0.0)	0·981 (0·970 to 0·992)	31·5 (27·5 to 36·4)	0·0 (0·0 to 0·0)	0·892 (0·872 to 0·912)	4220
Palliative	22·6 (19·6 to 25·6)	0.0 (0.0 to 0.0)	0·924 (0·910 to 0·939)	84·2 (81·6 to 86·9)	4·1 (2·7 to 5·5)	0·827 (0·817 to 0·838)	7345
Stage							
Distant stage	22·7 (19·9 to 25·6)	0.0 (0.0 to 0.0)	0·938 (0·926 to 0·951)	82·5 (80·0 to 85·1)	1·6 (0·8 to 2·5)	0·852 (0·842 to 0·861)	8531
Non-distant stage	10·2 (8·0 to 12·4)	0.0 (0.0 to 0.0)	0·936 (0·916 to 0·955)	57·3 (53·7 to 60·9)	0·1 (0·0 to 0·4)	0·874 (0·862 to 0·887)	7236
Cancer							
Breast	12·6 (9·1 to 16·1)	0.0 (0.0 to 0.0)	0·970 (0·956 to 0·983)	60·4 (55·2 to 65·6)	0·0 (0·0 to 0·0)	0·939 (0·928 to 0·951)	3408
Colon and Rectum	15·7 (11·5 to 19·9)	0.0 (0.0 to 0.0)	0·924 (0·898 to 0·949)	77·0 (72·1 to 81·9)	1·0 (0·0 to 2·2)	0·843 (0·827 to 0·860)	2868
Lung and Bronchus	23·3 (18·4 to 28·2)	0.0 (0.0 to 0.0)	0·916 (0·884 to 0·949)	84·8 (80·6 to 89·0)	3·9 (1·6 to 6·1)	0·820 (0·803 to 0·838)	2822
Ovary	12·2 (7·2 to 17·2)	0.0 (0.0 to 0.0)	0·956 (0·922 to 0·989)	67·7 (60·5 to 74·8)	1·2 (0·0 to 2·9)	0·895 (0·871 to 0·918)	1632
Hematological	18·8 (12·4 to 25·1)	0.0 (0.0 to 0.0)	0·943 (0·902 to 0·983)	49·3 (41·1 to 57·5)	0·7 (0·0 to 2·1)	0·819 (0·782 to 0·856)	1435
Skin	23·1 (13·7 to 32·4)	0.0 (0.0 to 0.0)	0·929 (0·897 to 0·962)	84·6 (76·6 to 92·6)	3·8 (0·0 to 8·1)	0·850 (0·816 to 0·884)	780
Head and Neck	6·8 (1·0 to 12·5)	0.0 (0.0 to 0.0)	0·970 (0·933 to 1·000)	66·2 (55·4 to 77·0)	0·0 (0·0 to 0·0)	0·839 (0·798 to 0·879)	739
Cervix Uteri	15·0 (6·0 to 24·0)	0.0 (0.0 to 0.0)	0·955 (0·906 to 1·000)	75·0 (64·0 to 86·0)	0·0 (0·0 to 0·0)	0·888 (0·847 to 0·929)	599
Urinary Bladder and Testis	9·1 (0·6 to 17·6)	0.0 (0.0 to 0.0)	0·960 (0·914 to 1·000)	77·3 (64·9 to 89·7)	2·3 (0·0 to 6·7)	0·853 (0·809 to 0·897)	433
Prostate	16·3 (5·2 to 27·3)	0.0 (0.0 to 0.0)	0·936 (0·849 to 1·000)	69·8 (56·0 to 83·5)	0·0 (0·0 to 0·0)	0·805 (0·751 to 0·858)	429
Brain and Other NS	27·5 (13·7 to 41·3)	0.0 (0.0 to 0.0)	0·892 (0·846 to 0·938)	97·5 (92·7 to 100·0)	12·5 (2·3 to 22·7)	0·821 (0·778 to 0·864)	399
Chemotherapy regimen							
Carbo/paclitaxel	10·0 (4·8 to 15·2)	0.0 (0.0 to 0.0)	0·940 (0·859 to 1·000)	50·0 (41·4 to 58·6)	0·8 (0·0 to 2·3)	0·846 (0·813 to 0·880)	1297
Folfox	13·9 (5·9 to 21·9)	0.0 (0.0 to 0.0)	0·952 (0·923 to 0·982)	68·1 (57·3 to 78·8)	0·0 (0·0 to 0·0)	0·863 (0·833 to 0·893)	719
Gemcitabine	29·1 (17·1 to 41·1)	0.0 (0.0 to 0.0)	0·865 (0·789 to 0·942)	7·0 (0·4 to 13·6)	0·0 (0·0 to 0·0)	0·992 (0·982 to 1·000)	547
Pemetrexed/carboplatin	25·0 (13·2 to 36·8)	0.0 (0.0 to 0.0)	0·960 (0·939 to 0·981)	81·8 (71·6 to 92·0)	3·6 (0·0 to 8·6)	0·807 (0·769 to 0·845)	518

© 2018 Elfiky AA et al. *JAMA Network Open.*

	30-day mortality, % (95% CI)			180-day mortality, % (95% CI)			
variable	top decile	bottom decile	AUC (95% CI)	top decile	bottom decile	AUC (95% CI)	validation sample size
Paclitaxel	17·6 (7·2 to 28·1)	0.0 (0.0 to 0.0)	0·989 (0·972 to 1·000)	75·0 (63·2 to 86·8)	7·7 (0·4 to 14·9)	0·738 (0·686 to 0·791)	505
Novel trial agents	8·6 (0·0 to 17·8)	0.0 (0.0 to 0.0)	0·942 (0·882 to 1·000)	77·1 (63·2 to 91·1)	0·0 (0·0 to 0·0)	0·870 (0·817 to 0·923)	343
Line of chemotherapy							
First line	14·9 (12·6 to 17·2)	0.0 (0.0 to 0.0)	0·941 (0·925 to 0·956)	62·2 (59·0 to 65·3)	0·1 (0·0 to 0·3)	0·865 (0·854 to 0·875)	9114
Subsequent line	20·6 (17·5 to 23·6)	0.0 (0.0 to 0.0)	0·938 (0·924 to 0·952)	83·8 (81·0 to 86·6)	0·8 (0·1 to 1·4)	0·864 (0·854 to 0·874)	6653
Year							
2012	16·8 (13·6 to 20·1)	0.0 (0.0 to 0.0)	0·951 (0·935 to 0·966)	76·2 (72·5 to 79·9)	0·4 (0·0 to 0·9)	0·887 (0·875 to 0·899)	5169
2013	17·5 (14·1 to 20·8)	0.0 (0.0 to 0.0)	0·935 (0·914 to 0·955)	80·1 (76·5 to 83·6)	0·0 (0·0 to 0·0)	0·869 (0·857 to 0·882)	4863
2014	18·5 (15·1 to 21·9)	0.0 (0.0 to 0.0)	0·928 (0·908 to 0·947)	73·8 (69·9 to 77·7)	0·2 (0·0 to 0·6)	0·837 (0·824 to 0·851)	4915
Age group							
21-40	10·4 (4·6 to 16·2)	0.0 (0.0 to 0.0)	0·963 (0·939 to 0·988)	71·7 (63·1 to 80·3)	0·0 (0·0 to 0·0)	0·901 (0·874 to 0·928)	1057
41-60	14·4 (11·6 to 17·1)	0.0 (0.0 to 0.0)	0·950 (0·935 to 0·966)	71·0 (67·4 to 74·5)	0·0 (0·0 to 0·0)	0·893 (0·883 to 0·903)	6263
61-80	19·8 (17·0 to 22·6)	0.0 (0.0 to 0.0)	0·936 (0·920 to 0·951)	79·3 (76·4 to 82·2)	1·4 (0·6 to 2·3)	0·850 (0·839 to 0·861)	7626
81-100	19·8 (11·1 to 28·4)	0.0 (0.0 to 0.0)	0·876 (0·806 to 0·947)	67·9 (57·7 to 78·1)	2·5 (0·0 to 5·8)	0·803 (0·768 to 0·838)	806
Gender							
Female	15·4 (13·2 to 17·7)	0.0 (0.0 to 0.0)	0·948 (0·935 to 0·962)	71·1 (68·3 to 73·9)	0·0 (0·0 to 0·0)	0·886 (0·877 to 0·894)	9968
Male	19·8 (16·6 to 23·1)	0.0 (0.0 to 0.0)	0·927 (0·910 to 0·944)	79·3 (76·0 to 82·6)	2·4 (1·2 to 3·7)	0·840 (0·828 to 0·852)	5799
Race							
White	17·2 (15·2 to 19·2)	0.0 (0.0 to 0.0)	0·940 (0·929 to 0·951)	74·9 (72·7 to 77·2)	0·2 (0·0 to 0·5)	0·870 (0·862 to 0·877)	13960
Non-white	17·7 (12·1 to 23·2)	0.0 (0.0 to 0.0)	0·941 (0·909 to 0·972)	76·2 (70·0 to 82·4)	0·0 (0·0 to 0·0)	0·870 (0·850 to 0·891)	1807
Insurance		0.0 (0.0 to 0.0)					
Private	16·1 (13·9 to 18·4)	0.0 (0.0 to 0.0)	0·949 (0·937 to 0·962)	73·2 (70·5 to 75·9)	0·0 (0·0 to 0·0)	0·886 (0·878 to 0·895)	10415
Medicare	19·3 (14·6 to 24·0)	0.0 (0.0 to 0.0)	0·938 (0·911 to 0·965)	84·1 (79·7 to 88·4)	2·2 (0·5 to 4·0)	0·854 (0·837 to 0·872)	2699
Medicaid	16·4 (7·1 to 25·7)	0.0 (0.0 to 0.0)	0·945 (0·914 to 0·976)	78·7 (68·4 to 89·0)	1·6 (0·0 to 4·7)	0·824 (0·784 to 0·865)	611
Self pay	33·3 (13·2	0.0 (0.0 to	0·932 (0·851	81·0 (64·2	4·8 (0·0 to	0·821 (0·753	210

© 2018 Elfiky AA et al. *JAMA Network Open.*

	to 53·5)	0.0)	to 1·000)	to 97·7)	13·9)	to 0·888)	

© 2018 Elfiky AA et al. *JAMA Network Open.*

eTable 2. Mean Mortality by Primary Cancer, Race, Sex, and Age Group for Distant Stage Disease From 18 SEER Registries

Selections:
Statistic type = Relative survival;
SEER registry = Eighteen SEER registries;
Year of diagnosis = 2006-2012;
Survival interval = 1-year;
Distant Stage

			**Esophagus**	**Stomach**	**Colon and Rectum**	**Liver and Intrahepatic Bile Duct**	**Pancreas**	**Larynx**	**Lung and Bronchus**	**Soft Tissue including Heart**	**Melanoma of the Skin**	**Breast**	**Cervix Uteri**	**Corpus and Uterus, NOS**	**Ovary**	**Prostate**	**Testis**	**Urinary Bladder**	**Kidney and Renal Pelvis**	**Brain and Other Nervous System**	**Thyroid**	**Hodgkin Lymphoma**	**Non-Hodgkin Lymphoma**	**Myeloma**
All races	Male and female	All ages	0.286	0.268	0.544	0.16	0.162	0.68	0.269	0.484	0.426	0.687						0.292	0.386	0.556	0.699	0.861	0.77	0.773
		00-64	0.319	0.311	0.667	0.179	0.225	0.728	0.33	0.562	0.456	0.758						0.383	0.457	0.652	0.817	0.923	0.831	0·861
		00-49	0.335	0.314	0.73	0.284	0.307	0.759	0.387	0.645	0.474	0.816						0.382	0.545	0.717	0.913	0.955	0.832	0.891
		00-44	0.369	0.318	0.734	0.379	0.371	0.721	0.429	0.664	0.46	0.834						0.431	0.603	0.725	0.948	0.964	0.834	0.904
		45-54	0.318	0.312	0.703	0.161	0.33	0.76	0.35	0.513	0.47	0.768						0.384	0.468	0.567	0.789	0.862	0.833	0.877
		50+	0.281	0.258	0.511	0.144	0.15	0.67	0.261	0.361	0.414	0.652						0.285	0.361	0.31	0·62	0·7	0.756	0.762
		55-64	0.314	0.307	0.621	0.154	0.203	0.712	0.312	0.387	0.445	0.715						0.376	0.412	0.379	0.717	0.804	0.829	0.846
		65+	0.247	0.224	0.418	0.132	0.118	0.598	0.23	0.314	0.386	0.583						0.243	0.31	0.228	0·55	0.583	0.708	0·71
		65-74	0.299	0.278	0.535	0.141	0.16	0.653	0.269	0.391	0.431	0.659						0.285	0.38	0.271	0.633	0.689	0.788	0.787
		75+	0.182	0.173	0.322	0.123	0.082	0.501	0.192	0.242	0.345	0.508						0.212	0.241	0.182	0.475	0.468	0.639	0.639
	Male	All ages	0.287	0.277	0.56	0.148	0.162	0.681	0.243	0.482	0.411	0.682				0.749	0.858	0.305	0.404	0.551	0.694	0.864	0.763	0.778

© 2018 Elfiky AA et al. *JAMA Network Open.*

		00-64	0.319	0.309	0.656	0.161	0.209	0.722	0.292	0.554	0.44	0.697				0.831	0.864	0.381	0.461	0.638	0.779	0.913	0.81	0.855
			**Esophagus**	**Stomach**	**Colon and Rectum**	**Liver and Intrahepatic Bile Duct**	**Pancreas**	**Larynx**	**Lung and Bronchus**	**Soft Tissue including Heart**	**Melanoma of the Skin**	**Breast**	**Cervix Uteri**	**Corpus and Uterus, NOS**	**Ovary**	**Prostate**	**Testis**	**Urinary Bladder**	**Kidney and Renal Pelvis**	**Brain and Other Nervous System**	**Thyroid**	**Hodgkin Lymphoma**	**Non-Hodgkin Lymphoma**	**Myeloma**
		00-49	0.335	0.317	0.701	0.245	0.291	0.756	0.348	0.655	0.462	0.722				0.835	0.879	0.394	0.522	0.711	0.864	0.946	0.811	0.884
		00-44	0.38	0.323	0.701	0.347	0.336	0.722	0.373	0.666	0.446	-				0.883	0.887	0.441	0.562	0.721	0.924	0.958	0.815	0.91
		45-54	0.317	0.315	0.689	0.137	0.223	0.764	0.311	0.497	0.446	0.607				0.838	0.792	0.401	0.477	0.492	0.717	0.836	0.808	0.871
		50+	0.282	0.27	0.534	0.135	0.151	0.673	0.237	0.338	0.399	0.676				0.747	0.666	0.3	0.384	0.29	0.639	0.719	0.75	0.767
		55-64	0.313	0.301	0.621	0.146	0.188	0.7	0.277	0.342	0.434	0.706				0.828	0.651	0.367	0.427	0.353	0.726	0.808	0.809	0.838
		65+	0.246	0.242	0.443	0.124	0.121	0.613	0.21	0.315	0.372	0.663				0.713	0.468	0.261	0.33	0·221	0.575	0.6	0.705	0.717
		65-74	0.289	0.291	0.537	0.132	0.152	0.662	0.241	0.38	0.407	0.703				0.788	-	0.302	0.391	0.327	0.648	0.703	0.774	0.787
		75+	0.187	0.186	0.342	0.113	0.086	0.522	0.176	0.243	0.338	0.621				0.661	-	0.226	0.259	0.096	0.489	0.467	0.637	0.644
	Female	All ages	0.281	0.251	0.528	0.194	0.16	0.674	0.299	0.488	0.457	0.687	0.483	0.503	0.691			0.263	0.353	0.562	0.702	0.856	0.779	0.767
		00-64	0.322	0.314	0.681	0.253	0.249	0.758	0.381	0.572	0.494	0.758	0.516	0.546	0.806			0.386	0.447	0.672	0.843	0.94	0.864	0.869
		00-49	0.333	0.309	0.763	0.395	0.33	0.771	0.43	0.632	0.501	0.817	0.542	0.597	0.823			0.363	0.593	0.724	0.94	0.97	0.871	0.901
		00-44	0.321	0.312	0.77	0.448	0.417	0.721	0.488	0.662	0.488	0.834	0.545	0.605	0.817			0.414	0.67	0.73	0.96	0.972	0.867	0.896
		45-54	0.321	0.306	0.72	0.273	0.249	0.744	0.398	0.529	0.527	0.77	0.536	0.534	0.825			0.351	0.439	0.693	0.842	0.917	0.874	0.886
		50+	0.277	0.238	0.487	0.168	0.149	0.658	0.291	0.387	0.446	0.652	0.45	0.489	0.668			0.255	0.317	0.332	0.607	0.674	0.763	0.755
		55-64	0.322	0.321	0.621	0.186	0.226	0.772	0.362	0.44	0.474	0.715	0.471	0.54	0.79			0.402	0.37	0.409	0.709	0.798	0.857	0.856
		65+	0.249	0.194	0.396	0.148	0.116	0.536	0.253	0.313	0.414	0.582	0.385	0.451	0.576			0.207	0.282	0.236	0.536	0.563	0.712	0.702
		65-	0.3	0.24	0.5	0.1	0.1	0.6	0.3	0.4	0.4	0.6	0.4	0.5	0.7			0.2	0.3	0.2	0.62	0.6	0.8	0.786

© 2018 Elfiky AA et al. *JAMA Network Open.*

		74	45	9	34	64	7	1	03	07	88	59	11	02	09			41	61	08	2	72	06	
		75+	0.168	0.157	0.308	0.135	0.079	0.412	0.208	0.241	0.359	0.507	0.35	0.38	0.452			0.189	0.221	0.266	0.468	0.469	0.641	0.635
			**Esophagus**	**Stomach**	**Colon and Rectum**	**Liver and Intrahepatic Bile Duct**	**Pancreas**	**Larynx**	**Lung and Bronchus**	**Soft Tissue including Heart**	**Melanoma of the Skin**	**Breast**	**Cervix Uteri**	**Corpus and Uterus, NOS**	**Ovary**	**Prostate**	**Testis**	**Urinary Bladder**	**Kidney and Renal Pelvis**	**Brain and Other Nervous System**	**Thyroid**	**Hodgkin Lymphoma**	**Non-Hodgkin Lymphoma**	**Myeloma**
White	Male and female	All ages	0.291	0.264	0.541	0.165	0.164	0.689	0.259	0.486	0.422	0.692						0.296	0.39	0.549	0.7	0.858	0.778	0.766
		00-64	0.33	0.306	0.673	0.19	0.23	0.747	0.321	0.57	0.451	0.774						0.386	0.47	0.654	0.828	0.931	0.847	0.86
		00-49	0.359	0.315	0.732	0.326	0.318	0.8	0.378	0.663	0.475	0.837						0.376	0.581	0.722	0.912	0.965	0.853	0.89
		00-44	0.397	0.322	0.735	0.451	0.38	0.788	0.412	0.679	0.458	0.852						0.433	0.65	0.731	0.946	0.973	0.854	0.9
		45-54	0.331	0.307	0.711	0.165	0.247	0.777	0.343	0.527	0.471	0.794						0.374	0.492	0.53	0.789	0.87	0.852	0.881
		50+	0.284	0.254	0.509	0.146	0.153	0.675	0.253	0.368	0.408	0.657						0.291	0.363	0.297	0.616	0.697	0.763	0.756
		55-64	0.322	0.298	0.628	0.16	0.206	0.727	0.305	0.395	0.435	0.727						0.386	0.417	0.405	0.743	0.817	0.84	0.845
		65+	0.247	0.223	0.417	0.133	0.122	0.598	0.223	0.326	0.383	0.587						0.25	0.31	0.211	0.535	0.579	0.715	0.706
		65-74	0.306	0.28	0.537	0.145	0.166	0.653	0.261	0.414	0.428	0.666						0.296	0.383	0.252	0.638	0.689	0.797	0.788
		75+	0.175	0.171	0.324	0.12	0.085	0.506	0.187	0.25	0.342	0.514						0.219	0.238	0.169	0.442	0.468	0.645	0.636
	Male	All ages	0.294	0.276	0.559	0.155	0.166	0.691	0.236	0.476	0.407	0.661				0.744	0.86	0.31	0.41	0.544	0.686	0.866	0.771	0.775
		00-64	0.332	0.308	0.661	0.173	0.215	0.743	0.284	0.554	0.436	0.717				0.835	0.867	0.383	0.475	0.642	0.776	0.927	0.825	0.856
		00-49	0.365	0.319	0.704	0.304	0.301	0.784	0.336	0.666	0.466	-				0.861	0.881	0.379	0.553	0.716	0.854	0.961	0.83	0.885

© 2018 Elfiky AA et al. *JAMA Network Open.*

		00-44	0.415	0.33	0.7	0.469	0.35	0.782	0.349	0.681	0.449	-				0.867	0.89	0.455	0.61	0.726	0.92	0.972	0.834	0.904
		45-54	0.334	0.311	0.697	0.146	0.238	0.781	0.304	0.496	0.446	-				0.854	0.793	0.395	0.5	0.459	0.701	0.853	0.827	0.871
		50+	0.286	0.269	0.534	0.138	0.154	0.68	0.23	0.344	0.393	0.654				0.741	0.675	0.305	0.389	0.279	0.629	0.718	0.757	0.766
			**Esophagus**	**Stomach**	**Colon and Rectum**	**Liver and Intrahepatic Bile Duct**	**Pancreas**	**Larynx**	**Lung and Bronchus**	**Soft Tissue including Heart**	**Melanoma of the Skin**	**Breast**	**Cervix Uteri**	**Corpus and Uterus, NOS**	**Ovary**	**Prostate**	**Testis**	**Urinary Bladder**	**Kidney and Renal Pelvis**	**Brain and Other Nervous System**	**Thyroid**	**Hodgkin Lymphoma**	**Non-Hodgkin Lymphoma**	**Myeloma**
		55-64	0.323	0.299	0.627	0.149	0.19	0.721	0.271	0.351	0.424	0.735				0.828	0.654	0.371	0.433	0.382	0.733	0.828	0.819	0.841
		65+	0.247	0.242	0.443	0.126	0.125	0.609	0.205	0.32	0.368	0.608				0.709	0.478	0.269	0.332	0.2	0.561	0.589	0.711	0.718
		65-74	0.295	0.295	0.538	0.134	0.159	0.656	0.236	0.403	0.404	0.658				0.792	-	0.318	0.396	0.309	0.642	0.692	0.783	0.793
		75+	0.181	0.184	0.347	0.116	0.087	0.53	0.173	0.233	0.331	0.556				0.656	-	0.231	0.258	0.08	0.463	0.462	0.64	0.646
	Female	All ages	0.276	0.241	0.522	0.193	0.163	0.676	0.287	0.501	0.454	0.693	0.498	0.52	0.699			0.268	0.352	0.556	0.709	0.845	0.788	0.755
		00-64	0.313	0.301	0.688	0.259	0.254	0.764	0.37	0.591	0.487	0.774	0.532	0.569	0.819			0.396	0.46	0.673	0.866	0.937	0.88	0.868
		00-49	0.32	0.308	0.764	0.387	0.343	0.858	0.423	0.659	0.497	0.838	0.556	0.622	0.838			0.37	0.64	0.73	0.945	0.971	0.894	0.898
		00-44	0.282	0.311	0.772	0.42	0.422	-	0.479	0.677	0.477	0.852	0.565	0.613	0.836			0.395	0.716	0.74	0.96	0.974	0.889	0.894
		45-54	0.312	0.297	0.73	0.255	0.262	0.76	0.389	0.561	0.531	0.795	0.541	0.567	0.835			0.334	0.47	-	0.859	0.907	0.892	0.895
		50+	0.272	0.226	0.483	0.17	0.152	0.647	0.279	0.399	0.443	0.657	0.466	0.505	0.677			0.26	0.314	0.322	0.607	0.668	0.771	0.744
		55-64	0.317	0.297	0.63	0.209	0.23	0.76	0.352	0.457	0.462	0.727	0.494	0.562	0.805			0.431	0.374	0.432	0.753	0.796	0.87	0.851
		65+	0.25	0.188	0.393	0.145	0.12	0.55	0.243	0.334	0.414	0.587	0.397	0.462	0.585			0.213	0.277	0.224	0.517	0.569	0.719	0.692
		65-74	0.366	0.243	0.536	0.172	0.175	0.641	0.293	0.433	0.486	0.666	0.414	0.515	0.726			0.242	0.36	0.179	0.634	0.685	0.815	0.781

© 2018 Elfiky AA et al. *JAMA Network Open.*

		75+	0.158	0.154	0.307	0.126	0.083	0.413	0.2	0.267	0.361	0.514	0.371	0.396	0.457			0.198	0.215	0.267	0.432	0.473	0.65	0.625
Black	Male and female	All ages	0.227	0.258	0.527	0.136	0.134	0.646	0.27	0.485	0.483	0.63						0.256	0.321	0.607	0.636	0.858	0.708	0.787
		00-64	0.231	0.3	0.617	0.148	0.179	0.664	0.31	0.531	0.491	0.678						0.361	0.345	0.669	0.738	0.874	0.728	0.854
			**Esophagus**	**Stomach**	**Colon and Rectum**	**Liver and Intrahepatic Bile Duct**	**Pancreas**	**Larynx**	**Lung and Bronchus**	**Soft Tissue including Heart**	**Melanoma of the Skin**	**Breast**	**Cervix Uteri**	**Corpus and Uterus, NOS**	**Ovary**	**Prostate**	**Testis**	**Urinary Bladder**	**Kidney and Renal Pelvis**	**Brain and Other Nervous System**	**Thyroid**	**Hodgkin Lymphoma**	**Non-Hodgkin Lymphoma**	**Myeloma**
		00-49	0.224	0.312	0.705	0.221	0.248	0.628	0.347	0.603	-	0.726						0.421	0.426	0.716	0.93	0.903	0.715	0.88
		00-44	0.264	0.294	0.716	0.28	0.304	0.441	0.381	0.621	-	0.765						-	0.465	0.713	0.958	0.916	0.72	0.898
		45-54	0.215	0.313	0.651	0.152	0.177	0.72	0.327	0.478	-	0.673						0.458	0.341	-	-	0.805	0.703	0.86
		50+	0.227	0.246	0.49	0.124	0.122	0.649	0.263	0.339	0.499	0.594						0.241	0.289	-	0.534	0.73	0.705	0.774
		55-64	0.237	0.293	0.561	0.128	0.165	0.649	0.295	0.337	-	0.63						0.293	0.287	-	0.584	0.72	0.758	0.843
		65+	0.219	0.207	0.4	0.099	0.088	0.602	0.228	0.288	0.473	0.522						0.169	0.28	-	0.489	0.659	0.66	0.715
		65-74	0.235	0.264	0.49	0.11	0.116	0.648	0.255	0.269	-	0.592						0.166	0.332	-	0.496	0.691	0.709	0.772
		75+	0.196	0.14	0.302	0.083	0.058	0.465	0.19	0.319	-	0.44						0.173	0.218	-	0.481	-	0.598	0.646
	Male	All ages	0.213	0.246	0.53	0.118	0.125	0.644	0.245	0.519	0.523	0.717				0.752	0.741	0.246	0.323	0.597	0.604	0.831	0.69	0.78
		00-64	0.21	0.279	0.604	0.13	0.149	0.649	0.28	0.543	0.485	0.645				0.813	0.752	0.353	0.341	0.623	0.749	0.832	0.706	0.843
		00-49	0.196	0.31	0.674	0.125	0.212	0.654	0.333	0.625	-	-				0.746	0.759	-	0.418	-	-	0.867	0.693	0.865
		00-	0.2	0.28	0.6	0.1	0.2	-	0.3	0.6	-	-				-	0.7	-	0.4	-	-	0.8	0.7	0.903

© 2018 Elfiky AA et al. *JAMA Network Open.*

		44	01	4	85	39	37		86	18							38		3			84		
		45-54	0.202	0.298	0.636	0.139	0.152	0.728	0.294	0.484	-	-				0.78	-	0.461	0.347	-	-	0.739	0.675	0.854
		50+	0.215	0.233	0.499	0.117	0.115	0.642	0.237	0.328	0.525	0.717				0.753	-	0.234	0.295	-	0.49	0.718	0.688	0.768
		55-64	0.216	0.265	0.557	0.124	0.139	0.62	0.265	0.301	-	-				0.822	-	0.293	0.301	-	-	0.67	0.74	0.825
		65+	0.218	0.2	0.407	0.07	0.086	0.632	0.203	0.385	-	-				0.707	-	0.139	0.287	-	0.364	0.795	0.64	0.703
		65-74	0.223	0.248	0.483	0.075	0.089	0.685	0.224	-	-	-				0.764	-	0.15	0.322	-	-	-	0.664	0.749
		75+	0.21	0.123	0.295	0.059	0.082	0.461	0.168	-	-	-				0.641		0.122	0.237	-	-	-	0.603	0.633
			**Esophagus**	**Stomach**	**Colon and Rectum**	**Liver and Intrahepatic Bile Duct**	**Pancreas**	**Larynx**	**Lung and Bronchus**	**Soft Tissue including Heart**	**Melanoma of the Skin**	**Breast**	**Cervix Uteri**	**Corpus and Uterus, NOS**	**Ovary**	**Prostate**	**Testis**	**Urinary Bladder**	**Kidney and Renal Pelvis**	**Brain and Other Nervous System**	**Thyroid**	**Hodgkin Lymphoma**	**Non-Hodgkin Lymphoma**	**Myeloma**
	Female	All ages	0.265	0.276	0.524	0.192	0.143	0.656	0.304	0.449	0.432	0.628	0.387	0.418	0.584			0.271	0.315	0.613	0.648	0.899	0.732	0.793
		00-64	0.293	0.337	0.633	0.223	0.223	0.721	0.357	0.517	-	0.679	0.412	0.438	0.676			0.378	0.353	0.705	0.733	0.942	0.765	0.866
		00-49	0.309	0.314	0.737	0.487	0.287	0.562	0.364	0.57	-	0.726	0.469	0.477	0.722			-	0.442	0.708	0.926	0.963	0.753	0.896
		00-44	-	0.305	0.747	-	0.362	-	0.376	0.626		0.764	0.466	0.537	0.718			-	0.515	0.699	0.97	0.964	0.754	0.892
		45-54	0.257	0.343	0.668	0.217	0.214	0.693	0.371	0.472	-	0.675	0.415	0.381	0.706			-	0.323	-	-	0.938	0.753	0.865
		50+	0.259	0.268	0.481	0.146	0.129	0.676	0.298	0.347	0.461	0.592	0.347	0.411	0.556			0.25	0.278	-	0.551	0.743	0.724	0.779
		55-64	0.298	0.356	0.566	0.142	0.204	0.783	0.345	0.365	-	0.629	0.365	0.445	0.641			0.293	0.253	-	0.556	0.805	0.783	0.862
		65+	0.223	0.217	0.394	0.144	0.089	0.488	0.257	0.213	-	0.517	0.323	0.395	0.476			0.203	0.27	-	0.533	0.566	0.678	0.724
		65-74	0.262	0.292	0.497	0.173	0.138	0.493	0.299	0.196	-	0.589	0.362	0.434	0.561			0.191	0.348	-	0.561	0.583	0.756	0.796
		75+	0.171	0.156	0.306	0.108	0.048	-	0.211	-	-	0.43	0.277	0.32	0.376			0.213	0.198	-	0.508	-	0.595	0.654

© 2018 Elfiky AA et al. *JAMA Network Open.*

© 2018 Elfiky AA et al. *JAMA Network Open.*

eTable 3. Model Performance by Category for Machine Learning vs RCT and SEER

**A**	**AUC (95% CI)**		
**Cancer and chemo regimen**	**ML**	**RCT**	**n**
Non-small cell lung adenocarcinoma: PEMETREXED/CARBOPLATIN	0.720 (0.659 to 0.782)	0.500 (0.500 to 0.500)	265
Colorectal adenocarcinoma: BEVACIZUMAB/OXALIPLATIN/5-FU/LV	0.860 (0.793 to 0.927)	0.500 (0.500 to 0.500)	151
Small cell lung carcinoma: ETOPOSIDE/CARBOPLATIN	0.672 (0.554 to 0.790)	0.500 (0.500 to 0.500)	82
Squamous cell carcinoma: CARBO/PACLITAXEL	0.816 (0.745 to 0.887)	0.500 (0.500 to 0.500)	139
Combined	0.771 (0.735 to 0.808)	0.555 (0.513 to 0.598)	637
**B**	**AUC (95% CI)**		
**Cancer**	**ML**	**SEER**	**n**
Lung and Bronchus	0.771 (0.750 to 0.791)	0.537 (0.511 to 0.563)	1,927
Breast	0.868 (0.850 to 0.887)	0.565 (0.534 to 0.595)	1,454
Colon and Rectum	0.794 (0.774 to 0.814)	0.563 (0.537 to 0.590)	1,780
Prostate	0.745 (0.688 to 0.802)	0.513 (0.448 to 0.578)	294
Combined	0.810 (0.799 to 0.822)	0.600 (0.585 to 0.615)	5,455

Note: *For each cancer-chemotherapy combination in* ***A****, we used the mortality rate reported in large randomized trials as the benchmark RCT mortality estimate; since these mortality rates do not vary by patient characteristics, they are only point estimates and thus form constant predictions. Constant predictions result in AUCs of 0.5*

© 2018 Elfiky AA et al. *JAMA Network Open.*

eResults. Code

```
## ------------------------------------------------------------------------ ##
## HEADER
## ------------------------------------------------------------------------ ##

## DESCRIPTION
## -------------------------------------------------------------- ##
# Creator: Maximilian J. Pany
# Date: 06/29/2017
# Language: R version 3.2.3

## TO-DO-LIST
## -------------------------------------------------------------- ##

## ------------------------------------------------------------------------ ##
## HEADER END
## ------------------------------------------------------------------------ ##


## ------------------------------------------------------------------------ ##
##  Preamble
## ------------------------------------------------------------------------ ##

## Cleaning house
## -------------------------------------------------------------- ##
rm(list=ls())
graphics.off()


## Libraries
## -------------------------------------------------------------- ##
# general
library(data.table)
library(xgboost)


## ------------------------------------------------------------------------ ##
## Data
## ------------------------------------------------------------------------ ##

# ! --------------- ! #
iteration <- "2016-09-11/"
outcome <- paste0("d180")
dth_outcome <- "dth_180d"
outcomePath <- paste0("project_path/pred/", iteration, outcome)
dir.create(outcomePath)
# ! --------------- ! #


## Load data
## -------------------------------------------------------------- ##
setwd("project_path/data")

cohort <- readRDS("cohort_chemo.Rds")
feat <- readRDS("all_features.Rds")
```

© 2018 Elfiky AA et al. *JAMA Network Open.*

```
## Split into training:testing sets (by years)
## -------------------------------------------------------------- ##
cohort[, year := format(as.Date(activation_dt), "%Y")]
cohort[, test := 0][year == "2012" | year == "2013" | year == "2014", test := 1]
overlap <- data.table(patient_id = intersect(cohort[test==0]$patient_id, cohort[test==1]$patient_id))
set.seed(2001)
overlap[, test := sample(c(0,1), .N, replace=T)]  # randomly assign overlapping pts to train or test
cohort.train <- cohort[test==0 & !(patient_id %in% overlap[test==1]$patient_id)]  # remove overlapping pts
from train cohort that assigned to test cohort
cohort.test <- cohort[test==1 & !(patient_id %in% overlap[test==0]$patient_id)]
cohort.train.overlap <- cohort[test==0 & (patient_id %in% overlap[test==1]$patient_id)]
cohort.train.overlap[, test := 1]  # reassign to test
cohort.test.overlap <- cohort[test==1 & (patient_id %in% overlap[test==0]$patient_id)]
cohort.test.overlap[, test := 0]  # reassign to train
cohort <- rbind(cohort.train, cohort.test.overlap, cohort.test, cohort.train.overlap)

# set outcome to be used
cohort[, outcome := get(dth_outcome)]

# feature files to: (a) generate or (b) load
trainRdsFile <- paste0(outcomePath, "/train.Rds")
trainXgbFile <- paste0(outcomePath, "/xgb.dtrain")
testRdsFile <- paste0(outcomePath, "/test.Rds")
testXgbFile <- paste0(outcomePath, "/xgb.dtest")

# train
if(!file.exists(trainXgbFile)){

  print("train files do not exist -- generating")
  train <- list()
  train$x <- merge(cohort[test == 0, .(outcome_id,test)], feat, by="outcome_id")
  train$x[, c("test", "outcome_id", "empi") := NULL]
  train$x <- as.matrix(train$x)  # needed for model
  train$y <- cohort[test == 0, outcome]
  train$id <- cohort[test == 0, outcome_id]
  saveRDS(train, file = trainRdsFile)
  dtrain <- xgb.DMatrix(data = train$x, label = train$y, missing=NA)
  xgb.DMatrix.save(dtrain, trainXgbFile)

} else{

  print("train files exist -- loading")
  train <- readRDS(file = trainRdsFile)
  dtrain <- xgb.DMatrix(trainXgbFile)

}

# test
if(!file.exists(testXgbFile)){

  print("test files do not exist -- generating")
  test <- list()
  test$x <- merge(cohort[test == 1, .(outcome_id,test)], feat, by="outcome_id")
  test$x[, c("test", "outcome_id", "empi") := NULL]
```

© 2018 Elfiky AA et al. *JAMA Network Open.*

```
  test$x <- as.matrix(test$x)  # needed for model
  test$y <- cohort[test == 1, outcome]
  test$id <- cohort[test == 1, outcome_id]
  saveRDS(test, file = testRdsFile)
  dtest <- xgb.DMatrix(data = test$x, label = test$y, missing=NA)
  xgb.DMatrix.save(dtest, testXgbFile)

} else{

  print("test files exist -- loading")
  test <- readRDS(file = testRdsFile)
  dtest <- xgb.DMatrix(testXgbFile)

}


## ---------------------------------------------------------------------- ##
##  Cross-validation
## ---------------------------------------------------------------------- ##

## Computation
## ------------------------------------------------------------ ##

# paras to CV: (1) number of trees, (2) tree depth, and (3) learning rate
cv.nround <- c(600,500,400,300)  # decreasing as depth increases -- map 1:1 to depth
cv.depth <- c(5,10,15,20)
cv.eta <- c(0.01, 0.05)  # multiply cv.nround by
cv.grid <- expand.grid(cv.depth, cv.eta)
cv.grid <- cbind(cv.grid, rep(cv.nround, length(cv.eta)))

# CV -- stratified (default)
set.seed(2002)
bst.cv <- list()
for (i in 1:nrow(cv.grid)) {
    print(paste0(i, ". iteration of ", nrow(cv.grid), " -- ", Sys.time()))
    cv <- xgb.cv(data = dtrain,
                 nfold = 4,
                 metrics = list("auc"),
                 max.depth = cv.grid[i, 1],
                 eta = cv.grid[i, 2],
                 nrounds = cv.grid[i, 3],
                 stratified = TRUE,
                 objective = "binary:logistic",
                 prediction = FALSE)
    bst.cv[[i]] <- cv
    bst.cv[[i]]$depth = cv.grid[i, 1]
    bst.cv[[i]]$eta = cv.grid[i, 2]
    bst.cv[[i]]$nround = seq(1, cv.nround[i], 1)
    saveRDS(bst.cv[[i]], file = paste0(outcomePath, "/bst_cv_round_", i, ".Rds"))

}


## Load distributed jobs
## ------------------------------------------------------------ ##
bst.cv <- list()
```

© 2018 Elfiky AA et al. *JAMA Network Open.*

```
for (i in 1:nrow(cv.grid)) {
  bst.cv[[i]] <- readRDS(paste0(outcomePath, "/bst_cv_round_", i, ".Rds"))
}
bst.cv <- rbindlist(bst.cv)
save(bst.cv, file = paste0(outcomePath, "/bst_cv.RData"))
saveRDS(bst.cv, file = paste0(outcomePath, "/bst_cv.Rds"))


## ----------------------------------------------------------------------- ##
##  Model fitting and prediction
## ----------------------------------------------------------------------- ##

## Find best parameters
## ------------------------------------------------------------- ##
# indx of max AUC
load(paste0(outcomePath, "/bst_cv.RData"))
max.auc.idx = which.max(bst.cv[, test.auc.mean])
max.auc <- bst.cv[max.auc.idx][, list(depth, eta, nround)]


## Fit the model on training data
## ------------------------------------------------------------- ##
set.seed(2003)
bst <- xgb.train(data = dtrain,
               max.depth = max.auc[, depth],
               eta = max.auc[, eta],
               nround = max.auc[, nround],
               subsample = 1,
               objective = "binary:logistic",
               eval.metric = "auc",
               verbose = 2)

save(bst, file = paste0(outcomePath, "/bst.RData"))
saveRDS(bst, file = paste0(outcomePath, "/bst.Rds"))


## Predict from the model in test data
## ------------------------------------------------------------- ##
yhat <- predict(bst, dtest)
save(yhat, file = paste0(outcomePath, "/yhat.RData"))
saveRDS(yhat, file = paste0(outcomePath, "/yhat.Rds"))


## ----------------------------------------------------------------------- ##
## FIN
## ----------------------------------------------------------------------- ##
```

© 2018 Elfiky AA et al. *JAMA Network Open.*

References

[1] Tianqi Chen, Tong He and Michael Benesty (2016). xgboost: Extreme Gradient Boosting. R package version 0.4-4. https://CRAN.R-project.org/package=xgboost

[2] The Elements of Statistical Learning. T. Hastie, R. Tibshirani, and J. Friedman. *Springer Series in Statistics*, *Springer New York Inc., New York, NY, USA,* (*2001*).

[3] R code for: (1) splitting data into derivation and validation sets, (2) training gradient boosted tree model, and (3) predicting mortality in the validation set (available at http://labsysmed.org/wp-content/uploads/2017/02/ChemoMortalityAnalysis.rtf).

[4] https://canques.seer.cancer.gov/cgi-bin/cq_submit?dir=surv2013&db=101&rpt=TAB&sel=1^0^^0^^^^0,3^1&y=Site^2,3,4,5,7,8,9,11,12,13,14,15,16,17,18,19,20,22,23,24,25,26&x=Race^0,1,2^Sex^0,1,2^Age%20at%20diagnosis^0,1,2,3,4,5,6,7,8,9&z=Stage%20at%20diagnosis^0,3&dec=1,0,1&template=null

[5] R Tibshirani (1996) Regression Shrinkage and Selection via the Lasso. *Journal of the Royal Statistical Society. Series B (Methodological)*. Vol. 58, No. 1 (1996), pp. 267-288

© 2018 Elfiky AA et al. *JAMA Network Open.*
